# Telerehabilitation for Knee Osteoarthritis in Brazil: A Feasibility Study

**DOI:** 10.5195/ijt.2020.6323

**Published:** 2020-12-08

**Authors:** Jéssica Bianca Aily, Christian John Barton, Stela Marcia Mattiello, Danilo De Oliveira Silva, Marcos De Noronha

**Affiliations:** 1 Department of Physical Therapy, Federal University of São Carlos, Center of Biological and Health Sciences, São Carlos, São Paulo, Brazil; 2 La Trobe University - School of Allied Health, Lasem Research Centre, Melbourne, Victoria, Australia; 3 La Trobe University – Rural Health School, Rural Department of Allied Health, Bendigo, Victoria, Australia

**Keywords:** Brazil, Knee, Osteoarthritis, Telerehabilitation

## Abstract

**Background:**

The effectiveness of telerehabilitation for a patient with knee osteoarthritis may depend upon the person's adherence to intervention. Thus, the aim of this study was to investigate whether people with knee osteoarthritis would adhere to exercise-therapy facilitated via multiple media in Brazil, a newly industrialized country.

**Method:**

This is a feasibility study, pre-post intervention. Middle aged (40-50 years) and elderly (≥70 years) people with knee osteoarthritis received in-person exercise-therapy instructions on the first day, along with a booklet and DVD (videos) to take home. Participants also received six motivational phone calls throughout the 12-week treatment. Satisfaction and adherence were assessed one week after intervention with the Exercise Adherence Rating Scale (EARS), sections B and C. Preference on the method used to adhere to exercises was recorded.

**Conclusion:**

Telerehabilitation was well accepted by middle-aged and elderly Brazilians with knee osteoarthritis. The preferred media to enhance adherence, was a booklet with descriptions of the exercises, especially for the elderly cohort.

Osteoarthritis (OA) is the most common chronic-degenerative joint disease in people of age 40 years and older (Arden & Leyland, 2013), and a leading cause of disability (Cross et al., 2014). The knee is one of the most affected joints by OA (Hunter & Bierma-zeinstra, 2019; Messier et al., 2013; Scopaz et al., 2009). This condition is present in 5-25% of people aged 60 years and older (Cross et al., 2014), and presents one of the greatest burdens in the health system globally (Cross et al., 2014). The pain and disability from OA can lead to reduced occupational capacity, physical activity, and a greater likelihood of co-morbidities (Cross et al., 2014).

Key clinical guidelines for knee OA recommend non-surgical and non-pharmaceutical strategies (Fernandes et al., 2013; McAlindon et al., 2014) with a focus on active management as the first line treatment. The latter includes exercise therapy, patient education, and weight loss (McAlindon et al., 2014). There is compelling evidence that exercise in particular reduces pain and improves function and quality of life. However, adherence to exercise-therapy is important, with long term adherence one of the biggest challenges for people with knee OA (Hong et al., 2008).

Exercise therapy for knee OA is typically prescribed and guided by physiotherapists via in-person interactions. However, access to such in-person healthcare can be limited by physical distance and costs (Fernandes et al., 2013), especially in rural areas (Hinman et al., 2017). The social distancing measures required by the COVID-19 global pandemic have also limited access to health professionals (Turolla et al., 2020). A potential solution to such accessibility challenges is telerehabilitation, which employs telecommunications technology to deliver rehabilitation across the entire acute, sub-acute, and community spectrum at a distance (Lee & Harada, 2013). A randomized controlled trial in people with knee and hip OA, compared usual physical therapy and a combined approach of in-person visits and Web-based physical activity intervention (i.e., e-exercise), and reported clinical improvements after 12 weeks in both groups (Kloek et al., 2018).

Health care models using telecommunication technology (e-health) such as video conferencing and even telephone use, can increase access to treatment and align with contemporary care models (Speerin et al., 2014). Findings from a clinical trial in the United Kingdom indicated that telephone services delivered by the National Health Service (i.e., initial assessment and exercises for acute and chronic musculoskeletal diseases) may be as effective as outpatient physiotherapy treatment, and provide faster and safer access to physiotherapy (Salisbury et al., 2014). Additionally, a systematic review reported that telephone interventions improved physical activity levels in adults managing various chronic diseases (Goode et al., 2012).

The successful implementation of telerehabilitation depends upon whether patients have access to the telecommunications technology, and are familiar with its operation (Dobson et al., 2016). It follows, therefore, that telerehabilitation outcomes may differ by country and socioeconomic status. For example, the United States, Norway, Japan and Australia score within the top 25 countries (of 139 countries) in the Networked Readiness Index (NRI), a tool to assess countries' preparedness to benefit from opportunities presented by the digital transformation and technologies (Balleret al., 2016). Countries with an upper-middle income economy, such as Brazil, are ranked in the lower half of the list. Importantly, differences in NRI may influence telerehabilitation's acceptability and outcomes (Allen et al., 2018; Azma et al.; Hinman et al., 2017). Countries ranked low in the NRI are likely to have gained access to computers and digital technology at a later stage, which may drive the observed age differences in the use of computers and technology in general (“Older Adults and Technology Use | Pew Research Center,” 2014). Even in the United States, a country highly ranked in NRI, people over 65 years make use of technology below the average for the country (“Older Adults and Technology Use | Pew Research Center,” 2014).

While countries with a higher income economy have been using telehealth for some time, countries such as Brazil have just started to consider this alternative due to the COVID-19 crisis (Dantas et al., 2020). Up until recently, telerehabilitation use was not even allowed by the Regulatory Agency responsible for the practice of Physiotherapy in Brazil, with a temporary exception authorized in March, 2020 (Resolution n° 516).

Therefore, the primary aim for this study was to investigate whether people with knee OA would adhere to an exercise therapy program delivered via multiple telerehabilitation options (DVD, web-based, telephone support), in Brazil, a country with an upper-middle income economy. Our secondary aims were to (1) analyse the effects of intervention on pain and function, and (2) compare acceptability of the telerehabilitation program by the two distinct age groups involved in the study: middle-aged (40 to 50 year of age) and elderly (70 years and over).

## METHODS

This mixed-methods study involved two parallel groups, pre- and post-test design and a qualitative design focused on identifying themes within the participants' experience of receiving telerehabilitation intervention. The study was approved by the local Human Research Ethics Committee (CAAE: 79229517.7.0000.5504). Recruitment was facilitated by inviting participants from another study (i.e., a non-interventional cross-sectional study). Informed written consent was provided by all participants.

### PARTICIPANTS

Twenty-nine participants (13 aged 40 to 50 years; and 16 aged > 70 years) were recruited from the community via radio, newspaper, and social media between April 2017 to November 2017. To be included participants had to meet the criteria of another concurrent study which aimed at evaluating the muscle architecture of middle-aged and older people with knee OA (Aily et al., 2019). Muscle architecture can be defined as the arrangement of muscle fibers relative to the axis of force generation (Lieber & Fridén, 2000). Inclusion criteria were age between 40 and 50 years (middle-aged) or ≥ 70 years (elderly), a BMI ≤ 30 kg/m2, and persistent pain in at least one knee for more than three months. Participants also had to be classified as grades II or III on the Kellgren and Lawrence (KL) scale (Kellgren & Lawrence, 1957), and meet the American College of Rheumatology criteria (ACR) (Altman et al., 1986). Exclusion criteria were previous trauma to lower limbs and/or knee ligament and meniscus injuries; participation in physical therapy in the previous six months; any previous lower limb surgery; or inability to comprehend and follow instructions as determined by de Almeida et al. (2018).

### INTERVENTION

At first day of intervention participants received individual and in-person instructions on how to perform each exercise, and how to follow the instructions included in a personal DVD and a booklet. Exercises were divided into lower body strengthening (knee flexors, extensors, bridge, step up and down), trunk exercises (plank), global exercises (exercises in standing position, involving whole body), and lower body stretching. Participants were expected to perform six different exercises per session, of which two were stretching (http://exercicio-joelho.trekeducation.org), at least 3 times a week. The exercise program lasted 12 weeks, with standardized bi-weekly difficulty progression (i.e., increase in number of repetitions or load). Instructions for the exercises could be accessed by the different media, as per the participants' preferences. Exercise delivery by telerehabilitation was asynchronous.

The participants' availability for regular phone calls was established in week 1. Participants received a phone call from an investigator (JA), for at least 10 minutes on weeks 2, 3, 4, 6, 8, and 11 to monitor their engagement and determine how often they were exercising. The phone calls were aimed to motivate participants to increase their adherence to the exercise program. The length of calls followed each participant's pace. They answered questions on how much exercise they had performed during the week and the timing of the exercise sessions. Questions also explored any difficulties in exercises, whether participants performed the exercises alone or with others, if the regimen had disrupted their day, and their preferences concerning the general exercise routine. The investigator was always sympathetic and encouraging.

Beginning in week 6, the material that had been provided to participants in the DVD was made available via a website link. The purpose was to gain information on patients' preferences for engaging with the exercise videos. They were able to choose between the DVD, internet, and booklet.

### PRE- AND POST-TEST DESIGN

Participants were assessed regarding satisfaction and adherence to the 12-week tele-rehabilitation exercises (primary outcome) one week after the end of treatment. Secondary outcomes included change in pain and function.

Adherence was assessed with the Brazilian-Portuguese version of the Exercise Adherence Rating Scale (EARS). EARS is a tool with three sections. Sections B and C are 5-point Likert questions (0 - completely agree to 4 - completely disagree), that assess self-reported adherence to home exercises (first 6 questions), and circumstances that help or hinder exercise compliance (10 questions). Some questions have the score inverted (1 and 4 in section B and 4, 5 and 6 in section C) to generate a maximal possible score of 24 for section B and 40 for section C. For both sections, higher scores represent better adherence (Newman-Beinart et al., 2017). Participants also answered six additional questions based on the study by Hinman et al. (2017) related to adherence, satisfaction, and adverse events (supplement material). Another three questions were presented to give participants the opportunity to describe positive and negative aspects of the telerehabilitation protocol.

Pain was assessed via the visual analogue scale (VAS; 0-100) (Bijur et al., 2001). Participants were asked about their worst pain in the previous week. Function was assessed via the Western Ontario and McMaster Universities Osteoarthritis Index (WOMAC) (Bellamy et al, 1988), a self-reported tool composed of 24 items divided into three subscales: pain, stiffness and physical function, in which higher scores (0-96) indicate a worse condition.

### STATISTICAL ANALYSIS

Data was presented descriptively through mean (SD), and percentages. Effect sizes (and 95% confidence intervals) were used to compare pre and post intervention for pain (VAS) and function (WOMAC) and to compare feasibility results (EARS) between cohorts (Table 2). Preference on how to inform the exercise (i.e., DVD, internet, booklet) was compared between cohorts via chi-square (n-1) test (Altman et al., 2013; Richardson, 2011), and within the cohorts via binomial tests (Howell, 2009) by comparing one of the preferences against the remaining two. The authors followed Cohen's (1988) schema to interpret the effect sizes (ES): 0.1 to 0.3: small effect; 0.3 to 0.5: moderate effect; 0.5 and higher: large effect.

### QUALITATIVE DESIGN

Six participants were randomly selected to participate in a focus group after completion of the 12-week intervention. The interview was semi-structured, following a topic guide (Appendix A) created by two of the investigators (CB and DOS). One investigator (JA) conducted the focus group interview, which lasted approximately 120 minutes. An inductive thematic analysis was used to generate themes from the qualitative data (Braun & Clarke, 2006). The audio recording was transcribed in Portuguese and then translated to English. First, one investigator (JA) independently read and re-read the transcript, then coded each transcript. Independently, a “framework” approach (Pope et al., 2006) was applied by one of the investigators, with previous experience in conducting and evaluating interviews (CB) (Barton et al., 2015; Neal et al., 2018). The thematic framework was formed by mapping the ideas and opinions stated by participants, and themes were generated. A second investigator (JA) also read through the transcript to reinforce the analysis. Codes remained close to participants' own words to capture their ideas. Trustworthiness of the qualitative data were determined by following the credibility, dependability, confirmability, and transferability criteria (Elo et al., 2014).

## RESULTS

Twenty-three of the 29 recruited participants completed the study. Two stopped attending the phone calls, one declined post-assessment, one moved to a different city, and two were unable to continue. Characteristics of participants are presented in Table 1.

**Table 1:** Characteristics of Participants

	All Participants	Middle Aged (n=10)	Older (n=13)
Age (y)	62.0 (15.1)	45.4 (2.4)	74.8 (2.9)
Age range (y)		40 to 50	70 to 80
Gender			
Women – n (%)	12 (52.2%)	7 (70%)	5 (38.5%)
Men – n (%)	11 (47.8%)	3 (30%)	8 (61.5%)
Unilateral Knee OA – n (%)	15 (65.2%)	5 (50%)	10 (76.9%)
Bilateral Knee OA – n (%)	8 (34.8%)	5 (50%)	3 (23.1%)
Height (m)	1.6 (0.1)	1.6 (0.1)	1.6 (0.1)
Weight (kg)	70.0 (12.1)	72.2 (11.4)	68.4 (12.9)
Body Mass Index (kg/m^2^)	26.6 (2.9)	26.5 (3.2)	26.7 (2.9)

*Note.* Data are presented as Mean (SD) where not otherwise indicated.

## ADHERENCE AND SATISFACTION

All participants reported good overall adherence to exercise (Tables 2 and 3). The average scores for EARS-B were 17.6 (5.2) out of 24. The middle-aged group had a mean EARS-B score of 17.0 (4.5) and the older group had a mean EARS-B score of 18.1 (5.6). For EARS-C, the total average score was 28.1 (6.3) out of 40. The middle-aged group had a mean EARS-C mean score of 26.5 (7.7) and the older group had a mean EARS-C score of 29.4 (4.6) (Table 2).

**Table 2:** EARS Outcomes for All Participants, and When Split into Middle-Aged and Older Cohorts

	All Participants	Middle Aged Participants (n=10)		Older Participants (n=13)		Effect Size for the difference between cohorts (95%CI)
EARS-B - Maximal possible score of 4 in each question (24 total)		Agree and Strongly Agree	Mean Score (SD)	Agree and Strongly Agree	Mean Score (SD)	
I do my exercises as often as recommended. *	3.1 (1.0)	70%	2.9 (1.0)	83%	3.3 (1.0)	0.4 (−.4 to 1.2)
I forget to do my exercises.	2.9 (1.4)	70%	2.7 (1.5)	75%	3.0 (1.3)	0.2 (−0.6 to 1.0)
I do less exercise than recommended by my healthcare professional.	2.3 (1.6)	50%	2.0 (1.6)	58%	2.5 (1.6)	0.3 (−0.5 to 1.1)
I fit my exercises into my regular routine. *	3.3 (1.1)	80%	3.3 (1.3)	83%	3.4 (1.0)	0.1 (−0.7 to 0.9)
I don't get around to doing my exercises.	2.8 (1.5)	80%	3.3 (1.3)	58%	2.5 (1.5)	−0.5 (−1.4 to 0.3)
I do most, or all, of my exercises.	3.2 (1.1)	70%	2.8 (1.3)	92%	3.5 (0.9)	0.6 (−0.3 to 1.4)
**Total Score**	**17.6 (5.2)**		**17.0 (4.5)**		**18.1 (5.6)**	**0.2 (−0.6 to 1.0)**
**EARS-C - Maximal possible score of 4 in each question (40 total)**						
I don't have time to do my exercises.	3.3 (1.1)	80%	3.0 (1.2)	92%	3.5 (0.8)	0.4 (−0.4 to 1.3)
Other commitments prevent me from doing my exercises.	2.9 (1.4)	60%	2.4 (1.6)	92%	3.2 (1.0)	0.6 (−0.2 to 1.5)
I don't do my exercises when I am tired.	2.7 (1.5)	70%	2.9 (1.4)	67%	2.5 (1.5)	−0.2 (−1.1 to 0.6)
I feel confident about doing my exercises. *	3.4 (0.9)	60%	3.0 (1.2)	92%	3.7 (0.6)	0.8 (−0.1 to 1.6)
My family and friends encourage me to do my exercises. *	3.0 (1.3)	50%	2.5 (1.4)	92%	3.3 (1.1)	0.7 (−0.2 to 1.5)
I do my exercises to improve my health. *	3.3 (1.2)	80%	3.1 (1.4)	83%	3.5 (0.9)	0.3 (−0.5 to 1.1)
I do my exercises because I enjoy them.	2.9 (1.2)	70%	2.9 (1.0)	67%	2.8 (1.3)	0.0 (−0.9 to 0.8)
I adjust the way I do my exercises to suit myself.	1.1 (1.3)	30%	1.4 (1.5)	17%	0.9 (1.1)	−0.4 (−1.2 to 0.5)
I stop exercising when my pain is worse.	2.2 (1.8)	50%	2.0 (1.8)	58%	2.3 (1.8)	0.2 (−0.6 to 1.0)
I'm not sure how to do my exercises	3.4 (1.0)	90%	3.3 (1.3)	92%	3.5 (0.7)	0.2 (−0.6 to 1.1)
**Total Score**	**28.1 (6.3)**		**26.5 (7.7)**		**29.4 (4.6)**	**0.5 (−0.4 to 1.3)**

*Note.* *Questions with scores inverted

**Table 3:** Call Frequency, Weekly Exercise Session and Personal Perception of Tele-Rehabilitation

All Participants		Middle Aged Participants (n=10)	Older Participants (n=13)
Number of calls	6.7 (0.7)	6.6 (0.5)	6.7 (0.8)
Average sessions per week	3.3 (1.6)	3.1 (1.1)	3.5 (1.8)
How satisfied are you with the exercise protocol followed up by phone calls? (0 to 7)	6.6 (0.7)	6.6 (0.8)	6.6 (0.6)
Did the videos help you to exercise regularly? (0 to 4)	3.1 (1.0)	3.7 (0.6)	2.6 (0.9)
Would you participate again in a telerehabilitation program? (YES/NO)	96% YES	100% YES	90% YES
What was your preferred way to follow the exercises?	n = 10/3*/16*	4/2/6	6/1/10*
(DVD/internet/Booklet) n and %	% = 43/13/70	40/20/60	46/8/77

*Note.* *binomial test - statistically significant when compared to remaining options within cohort.

Data from the additional six questions showed that participants only missed, on average, 16% of the calls, and were pleased to receive them (Table 3). They exercised on average over three times a week and felt the videos were helpful to maintaining regular exercise (Table 3). All but one participant would participate in telerehabilitation again if offered (Table 3).

Regarding adverse events, two participants reported personal problems that affected adherence. One reported difficulty in performing the exercises and two others mentioned knee pain as the reason for not exercising regularly.

Additionally, one participant expressed the preference for companionship during exercise. Participants' suggestions included the provision of equipment by the researchers (n=1), completing exercises in-person in groups instead (n=2), and provision of a more personalised approach with one to two weeks of in-person exercises before telerehabilitation (n=1).

### EFFECT OF INTERVENTION ON PAIN AND FUNCTION AND COMPARISONS BETWEEN MIDDLE-AGED AND ELDERLY COHORT

Improvement occurred for all participants (n=23) following the intervention for both pain (mean difference (MD), 95%CI = 2.5, 1.9 to 3.2; effect size (ES), 95%CI = −1.3, −2.1 to −0.5) and function (WOMAC MD, 95%CI = 15.7, 10.3 to 21.4; ES, 95%CI = −1.0, −1.8 to −0.2). No differences were identified when younger (n=10) and older (n=13) cohorts were compared.

### PREFERENCE OF INSTRUCTIONS (DVD, INTERNET, BOOKLET)

The booklet was the preferred media among all participants (p=0.0005), with a mean preference of 70% (95%CI 49% to 85%) and among the elderly cohort (p=0.002, mean preference 77%; 95%CI 49% to 93%). Internet was the least preferred option (all participants: p=0.045, mean preference 13%; 95%CI 3.7% to 32.98%). There were no other significant preferences within the different cohorts.

### QUALITATIVE FINDINGS

The themes generated were (1) Participants' perceptions about the outcome (including 2 subthemes); (2) Preferences; (3) Barriers; (4) Enablers; (5) Benefits and limitations of options provided; (6) Ongoing exercise or other options. A summary of themes and subthemes from patient's opinion is provided in table 4.

**Table 4:** Post Intervention Focus Group Qualitative Findings

Themes / Sub-themes	Quotes
1. Participant's perception about the outcome	
*1.1 Success/Happiness*	“*I thought it was great. As I already told you, I was thinking about replacing my knee with a prosthesis ….. but now it is better. Now I go up and down stairs, I am driving. To get inside the car, I needed to sit down and then, pull my leg on. Now, I do not need to do this anymore.*” “*I think that the most important were the exercises, because before I was very lazy, doing nothing. Now, I am feeling much better. In the first day of exercise, I was a little sore, then my body got used to it. It was very good.*” “*I do not have knee pain anymore*”
*1.2 Previous unsuccessful outcomes*	“*I did many other things before starting this project. I visited many medical doctors before coming to this project. They prescribed me lots of medicines, pills. Sometimes neighbours said to me "Wow, you are much better now.*” “*I visited a physiotherapist once a week. I attended 12 sessions. After that, I did 10 more sessions, and then, once again more 15 sessions. When I was there, he did some things… do you know? I went to my home better, without pain. However, in the next day, my knee was sore again. It means that the pain relief was only for a few hours.*”
2. Preferences	“*I followed the exercises through the booklet and the DVD, but I think that the DVD is better, because you can see the exercises*” “*We have access to Internet at home, but we did not access the exercises by the Internet or television (DVD) …. It was easier looking at the booklet.*” “*Inside the house I do not have DVD player, so I used the booklet ….. And by the Internet, I also do not have access, because I do not know how to use it ….. I used the booklet, because I did not have access to the DVD player… However, if I had access, it could be better than the booklet, because in the DVD I can watch the explanation about how to do the exercise, and in the booklet I can't.*”
3. Barriers	“*Laziness! I have it. But I don't know… Even with my laziness I did it. Sometimes, I thought "Oh, I will do it again…", but I did it. I think laziness was my main barrier.*” “*The hardest barrier for me was using the DVD because of my hearing loss.*” “*I had pain… In the beginning I had much pain, but I did the exercises slowly.*”
4. Enablers	*“We knew that you would call, so I thought like, "Oh, she is going to call" so, I had the commitment to do the exercises.”* *“If the exercises were done here at the university, this would be better for some people. I think at home was the best way for me. I did not have to come here and I could do the exercises anytime.”*
5. Benefits and limitations of options provided	*“Unless the face-to-face sessions occur periodically… to correct some exercises that we probably are doing wrong. Because with the telerehabilitation, if we are doing the exercises wrong, we will keep doing the exercises wrong, right?”* *“I think we do not need to come here every single time that we will do the exercises. There is no need to do this”* *“I guess nothing replaces the physiotherapist…… I could come here and say to you “look, I do this one and it hurts me,” then you would say “Let's change the exercise, let's do another one.” I do not know, something like this.”*
6. Ongoing exercise or other options	*“Sometimes I think that I do exercises even more than I should.”* *“The more you do, the better it is.”* *“Look, although I have a computer, I have never used Skype before. I do not know if I would have conditions to use it.”*

## DISCUSSION

To our knowledge, this is the first study to test an intervention for people with knee OA delivered via telerehabilitation in a newly industrialized country. Although the characteristics of Brazil may impose specific challenges for telerehabilitation, our results show good overall adherence, satisfaction, and acceptability of this method of exercise delivery. Importantly, feasibility of telerehabilitation was demonstrated in both middle-aged and elderly people, indicating the treatment may have wide reaching acceptance among people with knee OA.

Our results on adherence and satisfaction are similar to results reported from countries with a higher income economy (Choi et al., 2016; Nelson et al., 2017; Renda & Lape, 2018). Our findings align with those reported by Nelson et al. (2017), who investigated preferences in using technology to facilitate rehabilitation following total knee or hip replacement. Their online intervention, accessed via computer, tablet, or phone, showed older people feeling less comfortable in using the required technology, and preferring booklets to guide exercises. Current evidence indicates that it is important to consider that many older people seem to feel less comfortable with technology, regardless of their country of origin (Nelson et al., 2017; “Older Adults and Technology Use | Pew Research Center,” 2014). Interestingly, the current study also identified a clear preference towards booklets. Therefore, in Brazil, booklets should still be considered as an option for treatment reinforcement, particularly when combined with phone calls, following principles of evidence based practice, where a patient's preference needs to be considered (Herbert, 2011). Additionally, strategies to minimise this discomfort, such as hands-on training on technology, along with regular encouragement could be trialed to decrease resistance to technology.

Phone calls in our intervention seemed to have had a role in optimising adherence. Most participants were satisfied by being regularly called, and in most instances answered the phone calls (Table 3). Similar to previous studies in other settings (Goode et al., 2012; Hinman et al., 2017; Salisbury et al., 2014), this indicates that a phone conversation remains an effective way to maintain regular communication with patients and improve treatment adherence.

Our findings indicate large within participant improvement for both pain and function following telerehabilitation. However, these preliminary findings should be interpreted with caution, as this study did not contain a control group, and was not powered to compare cohorts. Nonetheless, pain reductions were approximately 2.5 points in a 10 point VAS, which is consistent with intervention arms in clinical trials, showing a reduction of two points in the VAS compared to a control group (Bellamy et al., 1992).

A positive response related to our intervention discerned from the focus group was the flexibility that telerehabilitation can offer. Participants were generally pleased they could choose the time that best fit their daily schedule to complete their exercise. One limitation shared by a participant was the lack of equipment provided to complete the exercises. Due to financial constrains for most participants, the acquisition of equipment was not viable. Therefore, participants were instructed to use any domestic objects instead of proper free weights (e.g., bags of beans, can of corn, etc.). Clinicians and researchers should consider financial constrains when implementing telerehabilitation that requires equipment, particularly when the population is of low socioeconomic status. One simple solution is to provide patients with the necessary equipment; although this would likely require additional funding. Alternative ways could be used to minimise costs, for example by using plastic water bottles in creative ways along with other low-cost material such as rubber balls and elastic bands. Furthermore, additional videos teaching participants to use objects found in the home could be used to help overcome exercise equipment cost barriers.

A reported point of dissatisfaction was the lack of companionship while performing exercises. Previous studies have shown that there is a potential relationship between companionship and adherence to exercise (Cavallo et al., 2014; Hong et al,, 2008; Marquez et al., 2016). Therefore, it is possible that, for some people with knee OA, telerehabilitation intervention may only be adhered to when alternative companionship (family, friends, other patients) can be arranged.

Key limitations for this study are the small sample size, the lack of a control group, and the short follow-up period. Furthermore, because the present study was a secondary analysis of a non-interventional cross-sectional study, there was a gap in the age bracket of participants (no participants between the ages 50 and 70), and all participants had a BMI of 30 or below and knee OA grades II and III in the KL scale. Also, two investigators were involved in interviewing participants and conducting the regular phone calls. Ideally a person not involved in the study would perform these tasks; due to lack of funding this limitation was unavoidable. Nevertheless, due to the nature of the study, which was related to feasibility (i.e., adherence and satisfaction), the authors believe this limitation did not have a significant impact on the study results.

In conclusion, participants in Brazil with knee OA adhered to an exercise regimen supported asynchronously by videos (i.e., DVD and web-based) and booklets, and monitored by phone calls. Booklets were the preferred supportive media to perform exercises, particularly in the older cohort, who were less comfortable using technology. It is important to understand the factors that influence adherence to exercise regimens, especially patients' preferred supportive media and any limitations related to their country of residence.

Cost savings, shorter waiting times, and reduced travel expenses will likely drive the increased adoption of telerehabilitation service delivery in newly industrialized countries such as Brazil. Progress could be accelerated by governmental actions that include investments in innovative technologies, training on the use of telehealth tools, and allowing wide population access to telerehabilitation.

## APPENDIX

### Topic Guide for the Semi-Structured Focus Group

1. What was the best way to access the exercises (booklet, DVD or internet) and why do you think this was the best way?
2. What was the reason that disturbed you most to do the exercises? (For example, I didn't use the internet because I don't have internet at home or I didn't use the DVD because I don't have time to watch, etc.).
3. What could we do for you to participate even more in the exercises?
4. In your opinion, can these delivery methods of treatment (booklet, DVD and internet) replace the face-to-face treatment supervised by a physical therapist? Why?
